# Risk factors and mortality of acute kidney injury within 1 month after lung transplantation

**DOI:** 10.1038/s41598-021-96889-1

**Published:** 2021-08-30

**Authors:** Nam Eun Kim, Chi Young Kim, Song Yee Kim, Ha Eun Kim, Jin Gu Lee, Hyo Chae Paik, Moo Suk Park

**Affiliations:** 1 Division of Pulmonary and Critical Care Medicine, Department of Internal Medicine, Institute of Chest Diseases, Severance Hospital, Yonsei University College of Medicine, 50-1 Yonsei-ro, Seodaemun-gu, Seoul, 03722 Republic of Korea; 2 Division of Pulmonary, Sleep and Critical Care Medicine, Department of Internal Medicine, Korea University Ansan Hospital, Korea University College of Medicine, Ansan, Republic of Korea; 3 Department of Thoracic and Cardiovascular Surgery, Severance Hospital, Yonsei University College of Medicine, Seoul, Republic of Korea; 4 Division of Pulmonary and Critical Care Medicine, Department of Internal Medicine, Ewha Womans University College of Medicine, Ewha Womans Seoul Hospital, Seoul, Republic of Korea

**Keywords:** Diseases, Medical research, Risk factors

## Abstract

After lung transplantation (LT), some patients are at risk of acute kidney injury (AKI), which is associated with worse outcomes and increased mortality. Previous studies focused on AKI development from 72 h to 1 week within LT, and reported main risk factors for AKI such as intraoperative hypotension, need of ECMO support, ischemia time or longer time on waiting list. However, this period interval rarely reflects medical risk factors probably happen in longer post-operative period. So, in this study we aimed to describe the incidence and risk factor of AKI within post-operative 1 month, which is longer follow up duration. Among 161 patients who underwent LT at Severance hospital in Seoul, Korea from October 2012 to September 2017, 148 patients were retrospectively enrolled. Multivariable logistic regression and Cox proportional hazard models were utilized. Among 148 patients, 59 (39.8%) developed AKI within 1-month after LT. Stage I or II, and stage III AKI were recorded in 26 (17.5%) and 33 (22.2%), respectively. We also classified AKI according to occurrence time, within 1 week as early AKI, from 1 week within 1 month was defined as late AKI. AKI III usually occurred within 7 days after transplantation (early vs. late AKI III, 72.5% vs 21.1%). Risk factor for AKI development was pre-operative anemia, higher units of red blood cells transfused during surgery, colistin intravenous infusion for treating multi drug resistant pathogens were independent risk factors for AKI development. Post-operative bleeding, grade 3 PGD within 72 h, and sepsis were more common complication in the AKI group. Patients with AKI III ([24/33] 72.7%) had significantly higher 1-year mortality than the no-AKI ([18/89] 20.2%), and AKI I or II group ([9/26] 34.6%), *log-rank test, P* < 0.001). AKI was associated with worse post-operative outcome, 3-month, and 1-year mortality after LT. Severity of AKI was usually determined in early post op period (ex. within 7 days) after LT, so optimal post-operative management as well as recipients selection should be considered.

## Introduction

Lung transplantation (LT) has become the standard treatment for patients with terminal lung diseases, and over 69,000 LTs have been performed worldwide from the early 90s^1^. The median survival of LT has improved recently to 6.7 years, compared to 5.6 years in the previous decade, as techniques and experiences with transplantation have grown^1,2^.

Acute kidney injury (AKI) arises as a consequence of different pathological conditions, such as renal hypoperfusion, nephrotoxic exposure, sepsis, or major surgery^3^. In LT, AKI is a common complication, shown to be associated with higher mortality and morbidity, primary graft dysfunction (PGD), and longer intensive care unit stay^4–6^.

The incidence of post-operative AKI after LT has been described literature, ranging from 9.4 to 68.8%^4–10^. This wide range could be explained by the use of different AKI definitions, and recently, Ploypin et al*.* reported that the overall estimated incidence rate of AKI after LT is 52.5% via a systematic review adjusting for different AKI definitions such as the Risk, Injury, Failure, Loss of function; and End-stage kidney disease (RIFLE) criteria and the Acute Kidney Injury Network (AKIN) criteria^11^.

Another reason for the wide range of incidence rates could be the timing at which AKI is determined. Some studies have defined AKI as that within 72 h after LT, as a concept called early post-operative AKI^8^, others have defined AKI as that occurring over a post-operative period of 3–14 days^5,12,13^.

In the present study, AKI was defined as that within 1 month after LT, longer than previous studies. We did this to include factors that have rarely been documented, but may pose greater susceptibility to AKI, such as infections with antimicrobial resistance species, and increasing exposure to nephrotoxic agents. We also compared risk factors, post-operative outcomes, including 3-month and 1-year mortality, according to AKI severity and timing of AKI development.

## Materials and methods

### Study population and data collection

**Figure 1:**
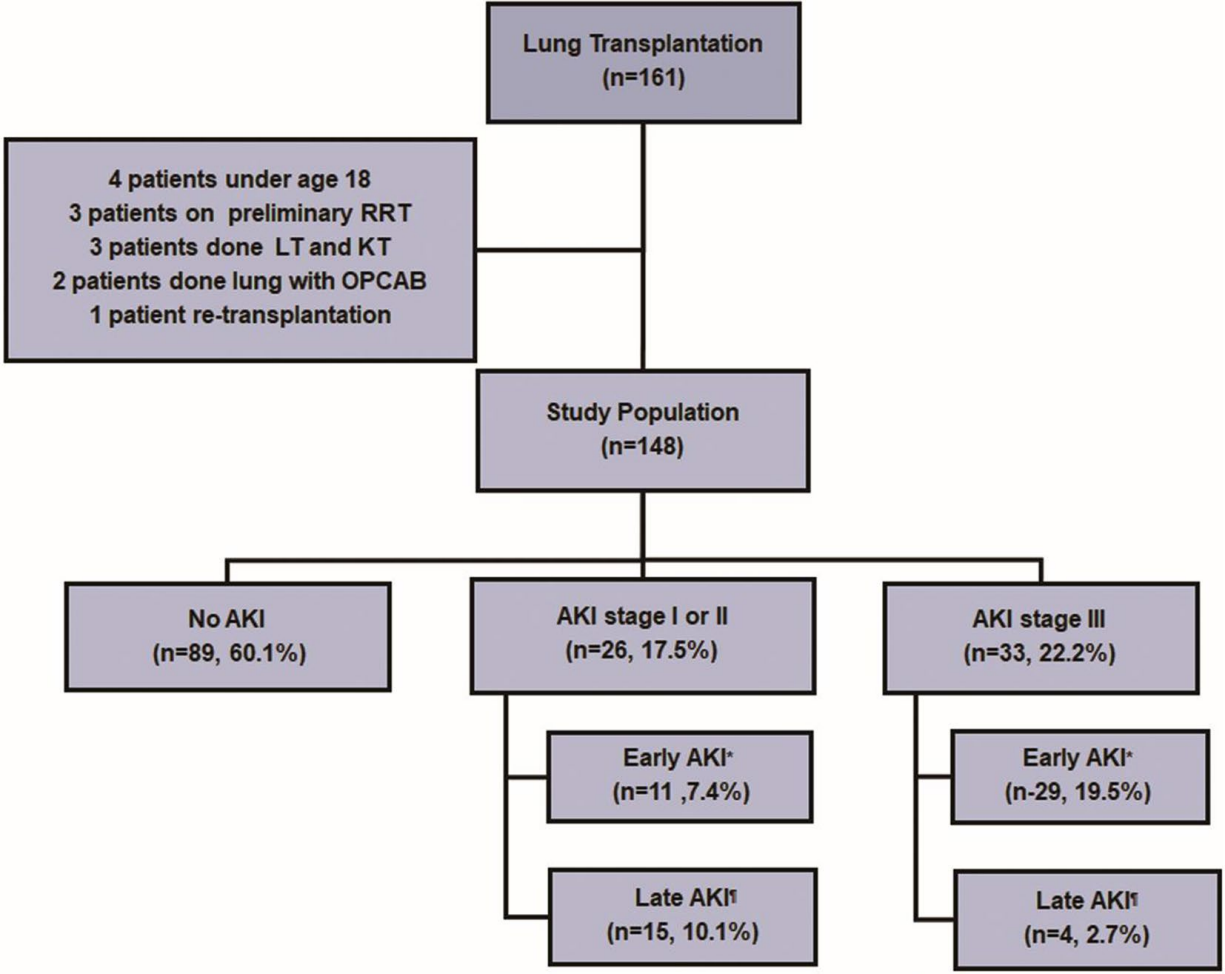
Study population and incidence of acute kidney injury of lung transplant recipients**.** 39.9% recipients developed acute kidney injury after lung transplantation within 1 month. *Early AKI, acute kidney injury within 1 week; ^**¶**^late AKI, acute kidney injury after 1 week but within 1 month; *RRT* renal replacement therapy, *LT* lung transplantation, *KT* kidney transplantation, *OPCAB* off-pump coronary artery bypass, *AKI* acute kidney injury.

We retrospectively reviewed the medical records of 161 LT recipients’ treated at Severance Hospital, in Seoul, South Korea from October 2012 to September 2017. Patients were excluded if they were under the age of 18 years, underwent both lung and kidney transplantation, underwent LT with off-pump coronary artery bypass, had already undergone renal replacement therapy(RRT), or had undergone re-transplantation. Thirteen patients were excluded (Fig. 1).

Data on variables related to baseline patient characteristics, pre-operative and post-operative outcomes, and survival status were obtained from the hospital medical records. Donor data were collected from the Korean Network of Organ Sharing database.

The primary outcome was the incidence of AKI within 1 month in patients that underwent LT. The AKIN definition was used to grade the severity of AKI. Secondary outcomes were days of mechanical ventilator usage, length of stay (LOS) in the intensive care unit (ICU), and 3-month and 1-year mortality.

The Institutional Review Board of Severance Hospital approved the study protocol in accordance with guidelines of the Declaration of Helsinki (IRB No: 4-2020-0228) and waived the need to obtain informed consent considering the retrospective nature of the study. All methods were carried out in accordance with relevant guidelines and regulations.

### Immunosuppression protocol and post-operative fluid balance

All patients received intravenous methylprednisolone (500 mg) intraoperatively just before the reperfusion of each graft. Post-operatively, patients received a 7-day tapered course of prednisolone, followed by oral prednisolone 0.5 mg/kg/day tapered to 0.15 mg/kg/day over 6 months. Triple immunosuppression therapy, such as prednisolone, tacrolimus, and mycophenolate mofetil, were used to maintain immunosuppression after transplantation. Cefepime and teicoplanin were used as routine prophylactic antibiotics after LT. Ganciclovir, itraconazole, and sulfamethoxazole/trimethoprim were used as routine universal prophylactic therapy.

In the immediate post-operative period, most patients were titrated a daily average of 500 ml of negative fluid balance, based on the amount of body weight gain after surgery. Input and output control was slowly modulated in case of AKI or shock status using inotropics.

### AKI definition

AKI was evaluated in accordance with AKIN classification. AKI stage I was defined as an increased serum creatinine (sCr) level ≥ 0.3 mg/dl (≥ 26.5 μmol/l) or an increase in sCr of 150–200% (1.5- to 2- fold) above baseline, along with a urine output of < 0.5 ml/kg/h for more than 6 h. AKI stage II was defined as an increase in sCr of 200% to 300% (> 2-to 3-fold) above baseline, with a urine output of < 0.5 ml/kg per hour for more than 12 h. AKI stage III was defined as an increase in sCr of more than 300% (> 3-fold) above baseline, or (sCr) level ≥ 4.0 mg/dl (≥ 35.4 μmol/l), with an acute increase of at least 0.5 mg/dl (44 μmol/l) or placement on RRT and urine output < 0.3 mg/kg/h for 24 h or anuria for 12 h^14^.

Baseline sCr levels were evaluated before LT. If AKI developed in a patient, we tracked the data daily until the self-remission of AKI. We obtained sCr levels immediately after LT up until 1 month after surgery. The modification of diet in renal disease equation was utilized to obtain the baseline estimated glomerular filtration rate (eGFR). Pulmonary hypertension was defined as the mean pulmonary artery pressure ≥ 25 mm Hg at rest, measured during right heart catheterization^15^.

Early AKI was defined as AKI that developed within 1 week after LT. Late AKI was defined as AKI that developed after 1 week, but within 1 month, after LT.

According to previous literatures reported in Asian transplantation center, they commonly use AKI definition as AKIN, and MDRD equation. So we decided to use the same modality^13,16^.

### Post-operative complications

Primary graft dysfunction was graded according to the criteria of the International Society of Heart and Lung Transplant (ISHLT) Working Group. The proposed standardized definition of PGD was based on diffuse pulmonary edema in an allograft on a chest radiograph and a PaO_2_/FiO_2_ (P/F) ratio^17^.

Pneumonia was defined as the presence of infiltration on chest X-ray and positive sputum cultures on bronchioalveolar lavage cultures requiring antibiotic treatment within 1 month. Sepsis was defined as any positive blood culture within 1 month after LT, with evidence of organ dysfunction^18^.

Operation time was defined as duration between induction time to end of skin suture time. Ischemia time was defined as the time interval between the application of the aortic cross-clamp during harvesting and reperfusion of the graft in the recipient.

### Statistical analysis

Most data were analyzed using IBM SPSS Statistics for Windows, version 23 (IBM Corp., Armonk, N.Y., USA). Data are expressed as mean or median values with standard deviations or interquartile ranges (IQRs). Continuous variables are presented as median (IQRs) were analyzed using the Mann–Whitney U test. Categorical variables are displayed as frequency distributions (n) and simple percentages (%), and were analyzed using the chi-square test or Fisher’s exact test. Binary logistic regression analysis was performed to identify risk factors associated with AKI development within 1 month. Kaplan–Meier curves stratified according to AKI stage were constructed for the evaluation of mortality at 3-months and 1-year after LT. Factors associated with survival were adjusted for using Cox regression analysis. Multivariable Cox proportional hazards analyses, including all variables with *P* less than 0.1 on univariate analysis, were performed to determine variables associated with long-term mortality. To assess the validity of the proportional hazards assumption, log-minus log-survival function, time-dependent covariate analysis was used. The time-dependent covariate was not statistically significant, suggesting that the proportional hazards assumption was reasonable. Estimates for hazard ratios (HR) and 95% confidence intervals (CIs) were reported. For all analyses, *P* values less than 0.05 were considered statistically significant.

## Results

### Characteristics and incidence of post-operative AKI

A total of 148 lung transplants recipients were analyzed. Among them, 59 patients (39.9%) developed AKI within 1 month; 89 patients (60.1%) maintained normal renal function. Twenty-six patients (17.5%) were classified with AKI stage I or II and 33 recipients (22.2%) were classified with AKI stage III. Among patients with AKI stage III, 88% (29/33) developed AKI within 1 week after LT during the early post-operative period. Meanwhile, the majority of patients with AKI stage I or II had increased sCr levels after 1 week and within 1 month after LT (Fig. 1).

There were no significant differences in demographic characteristics, such as age or sex, between the AKI and the no-AKI groups (Table 1). However, pre-operative comorbidities, including bridging mechanical ventilation (MV) and Extra corporeal membrane oxygenation (ECMO) for respiratory failure prior to surgery, were more severe in the AKI group. Pre-operative anemia and hypoalbuminemia was observed in the post-operative AKI group.

**Table 1:** Baseline characteristics of 148 recipients according to acute kidney injury within 1 month after lung transplantation.

	No AKI group (n = 89)	AKI group (n = 59)	*P* value^a^
Age, years, mean ± SD	53.13 ± 11.50	55.24 ± 11.13	0.272
Male, sex, n (%)	55 (61.8)	34 (57.6)	0.610
BMI, kg/m^2^, mean ± SD	21.62 ± 3.88	21.30 ± 3.96	0.625
**Primary lung disease, n (%)**			0.307
IPF	55 (67.9)	26 (44.1)	
CTD related ILD	11 (12.5)	10 (16.9)	
AIP	1 (25.0)	3 (5.1)	
BO after PBSCT	6 (6.8)	6 (10.2)	
Other*	15 (17.1)	14 (23.8)	
**Comorbidities, n (%)**			
Hypertension	25 (28.1)	11 (18.6)	0.190
Diabetes mellitus	23 (25.8)	15 (25.4)	0.954
Pulmonary hypertension	43 (48.3)	30 (50.8)	0.763
**Laboratory test, mean ± SD**			
Hb, g/day	12.19 ± 2.55	11.10 ± 2.25	0.009
Cr (baseline), mg/dL	0.57 ± 0.17	0.53 ± 0.23	0.170
eGFR (baseline)	88.8 ± 3.91	88.9 ± 3.92	0.879
Albumin, mg/day	3.17 ± 0.83	2.89 ± 0.74	0.043
**Donor data**			
Donor age, years, mean ± SD	41.30 ± 12.00	42.2 ± 12.40	0.664
Donor sex, n (%)	55 (56.1)	32 (62.7)	0.149
Smoking, pack years, mean ± SD	6.52 ± 11.30	7.78 ± 10.90	0.501
**Perioperative variables**			
Pre-op ECMO bridging, n (%)	10 (11.2)	14 (23.7)	0.044
Pre-op MV, n (%)	17 (19.1)	23 (39.0)	0.008
pre-op APACHE II score, mean ± SD	24.25 ± 6.60	25.42 ± 8.12	0.639
**Intra-operative support, n (%)**			0.453
CPB	3 (3.4)	3 (5.1)	
ECMO	86 (96.6)	56 (94.9)	
**Lunt transplantation**			0.599
Bilateral	81 (94.2)	54 (94.7)	
Single	5 (5.8)	3 (5.3)	
Ischemia time, h, mean ± SD	306.42 ± 89.44	311.90 ± 83.58	0.708
Operation time, h, mean ± SD	7.78 ± 1.29	8.55 ± 1.95	0.005
Donor-recipient size mismatch, n (%)	17 (19.1)	13 (22.0)	0.664
Number of RBC transfusion units, n, mean ± SD	6.30 ± 3.93	9.00 ± 5.50	0.002
Hemodynamic unstable event during surgery, n (%)	56 (69.1)	39 (73.6)	0.361
Intraoperative fluid intake, ml	6681.30 ± 351.8	8874.70 ± 548.7	0.001
Intraoperative fluid output, ml	1412.86 ± 141.39	1995.38 ± 228.05	0.033
**Postoperative antibiotics, n (%)**			
Colistin intravenous infusion	13 (14.6)	28 (47.5)	< 0.001
Colistin inhalation	26 (29.2)	27 (45.8)	0.040
Amikacin intravenous infusion	12 (13.5)	11 (18.6)	0.396
Amphotericin B intravenous infusion	12 (13.5)	14 (23.7)	0.109
Renal replacement therapy, n (%)	0 (0)	21 (35.6)	0.001
3-month mortality	4 (4.5)	13 (22.0)	0.001
1-year mortality	18 (20.2)	33 (55.9)	0.001

Data are presented as numbers (%) or means ± standard deviation (SD).

*BMI* body mass index, *IPF* idiopathic pulmonary fibrosis, *CTD* connective tissue disease, *ILD* interstitial lung disease, *AIP* acute interstitial pneumonia, *BO after PBSCT* bronchiolitis obliterans after peripheral blood stem cell transplantation, *Egfr* estimated glomerular filtration rate, *Hb* hemoglobin, *Cr* creatinine, *ECMO* extra corporeal membrane oxygenation, *CPB* cardiopulmonary bypass, *MV* mechanical ventilation, *RBC* red blood cell, *APACHE* acute physiology and chronic health evaluation.

*Other group included bronchiectasis, cor pulmonale, lymphangioleiomyomatosis (LAM), chronic obstructive pulmonary disease (COPD), and steroid resistant bronchiolitis obliterans organizing pneumonia.

^a^*P* value determined by *t* test or χ^2^ test.

### Intra-operative variables and donor characteristics

During LT, operation times were significantly longer in the AKI group than in the non-AKI group, and a higher number of packed red blood cell (RBC) units was transfused during surgery. Larger fluid intake and output was observed in the AKI group during surgery. Ischemia time and size mismatch between the recipient and donor lungs were similar between the groups. There was no significant difference in hemodynamic instability (systolic blood pressure under 90 mmHg or vasopressor use) during transplantation. Over 90% of the patients in the AKI and non-AKI groups underwent bilateral lung transplantation. Most recipients received ECMO support for intraoperative circulatory support than cardio-pulmonary bypass (CPB), Donor variables, such as age, sex, and smoking pack years, were similar in the two groups.

### Post-operative variables

In the post-operative period, treatment with intravenous colistin or by inhalation was significantly higher in the AKI group. Use of other nephrotoxic agents such as amphotericin B and amikacin, were similar between the two groups. Among patients with AKI, 35.6% received renal replacement therapy due to kidney failure, which was significantly higher than that in the non-AKI group.

### Univariable and multivariable analysis for AKI

Risk factors associated with AKI after LT are shown in Table 2. Univariate analysis revealed that pre-operative anemia, number of RBC transfusion units, usage of intravenous colistin or by inhalation, and bridging MV or ECMO were risk factors for AKI. After multivariable analysis, anemia, number of RBC transfusion units during surgery, and intravenous colistin infusion were independent risk factors for AKI. Application of bridging ECMO or MV before LT was not associated with post-operative AKI.

**Table 2:** Logistic regression of risk factors for AKI after lung transplantation.

Variables	Univariate		Multivariable	
	OR (95% CI)	*P* value^a^	OR (95% CI)	*P* value^a^
Age	1.01 (0.98–1.04)	0.27		
Sex, male	1.19 (0.60–2.32)	0.61		
BMI (kg/m^2^)	0.97 (0.89–1.06)	0.62		
**Primary lung disease**				
IPF	0.49 (0.25–0.95)	0.04	0.536 (0.245–1.171)	0.118
CTD related ILD	1.44 (0.57–3.66)	0.43		
AIP	4.71 (0.48–46.45)	0.18		
BO after PBSCT	1.57 (0.48–5.11)	0.46		
Hypertension	0.58 (0.26–1.30)	0.19		
Diabetes mellitus	0.97 (0.46–2.07)	0.95		
Pulmonary hypertension	1.10 (0.57–2.13)	0.76		
Pre-op APACHE II score	1.02 (0.94–1.12)	0.63		
**Laboratory findings**				
Hb (g/day)	0.83 (0.72–0.95)	0.01	0.836 (0.701–0.996)	0.045
Cr (baseline), mg/dL	0.30 (0.05–1.67)	0.17		
eGFR	1.00 (0.92–1.09)	0.87		
Albumin (mg/day)	0.64 (0.42–0.99)	0.21		
**Perioperative variables**				
Operation time	1.35 (1.08–1.70)	0.11		
Unit number of RBC transfusion	1.13 (1.04–1.21)	0.001	1.109 (1.018–1.208)	0.018
ECMO bridging	2.46 (1.0–5.9)	0.04	0.879 (0.246 -3.139)	0.879
Pre-op MV	2.7 (1.28–5.69)	0.009	1.544 (0.478–4.987)	0.468
Ischemia time	1.00 (0.99–1.00)	0.72		
Size mismatch	1.20 (0.53–2.69)	0.66		
Intra op CPB	1.53 (0.29–7.88)	0.60		
Hemodynamic instablity	1.24 (0.57–2.69)	0.58		
**Post**-**operative variables**				
Colistin intraveous infusion	5.28 (2.42–11.51)	0.001	4.346 (1.695–11.144)	0.002
Colistin inhalation	2.04 (1.03–4.06)	0.04	0.679 (0.265–1.736)	0.679
Amikacin intravenous-infusion	1.47 (0.60–3.59)	0.39		
Amphotericin B intravenous-infusion	1.20 (0.85—4.69)	0.11		
Donor age	1.00 (0.97–1.03)	0.66		
Donor sex	1.00 (0.55–2.11)	0.80		
Donor smoking	1.00 (0.98–1.0)	0.49		

^a^*P* value determined by logistic regression model.

*BMI* body mass index, *IPF* idiopathic pulmonary fibrosis, *CTD* connective tissue disease, *ILD* interstitial lung disease, *AIP* acute interstitial pneumonia, *BO after* *PBSCT* Bronchiolitis obliterans after peripheral blood stem cell transplantation, *eGFR* estimated glomerular filtration rate, *Hb* hemoglobin, *Cr* creatinine, *ECMO* extra corporeal membrane oxygenation, *MV* mechanical ventilation, *RBC* red blood cell, *APACHE* Acute Physiology and Chronic Health Evaluation.

### Association between AKI and other post-operative complications

We compared the occurrence of other post-operative complications between AKI and the no-AKI group. Post-operative bleeding, grade 3 PGD, and sepsis developed more commonly in the AKI group. AKI was associated with longer length of stay in the ICU and prolonged ventilator support during weaning (Table 3).

**Table 3:** Postoperative complications among lung transplant recipients according to AKI.

	No AKI group (n = 89)	AKI group (n = 59)	*P* value^a^
Postoperative bleeding, n (%)	4 (4.5)	17 (28.8)	0.001
GI bleeding, n (%)	8 (9.0)	7 (11.9)	0.381
Grade 3 PGD, 72 h, n (%)	8 (9.0)	16 (27.1)	0.004
Sepsis, n (%)	15 (16.9)	22 (37.3)	0.005
Pneumonia, n (%)	10 (38.5)	19 (57.6)	0.116
ICU stay, day (IQR)	7.00 (5.00–12.00)	14.00 (6.00–28.00)	0.004
MV day, day (IQR)	5.00 (4.00–10.00)	14.00 (6.00–26.50)	0.001

Data are presented as number (%) or medians (interquartile ranges).

*GI bleeding* gastrointestinal bleeding, *PGD* primary graft dysfunction, *ICU* intensive care unit, *MV* mechanical ventilation.

^a^*P* value determined by *t* test or χ^2^ test.

Stratified by AKI stage, grade 3 PGD and sepsis occurred more commonly in patients with AKI stage III. Also, prolonged ventilator support was more common in the AKI III group (Table 4).

**Table 4:** Postoperative complications among lung transplant recipients according to AKI severity.

	AKI stage I or II (n = 26)	AKI stage III (n = 33)	*P* value^a^
Postoperative bleeding, n (%)	5 (19.2)	12 (36.4)	0.124
GI bleeding, n (%)	2 (7.7)	5 (15.2)	0.323
Grade 3 PGD, 72 h, n (%)	3 (11.5)	13 (39.4)	0.016
Sepsis, n (%)	3 (11.5)	19 (57.6)	0.001
Pneumonia, n (%)	5 (19.2)	12 (36.4)	0.124
ICU Stay, day, median (IQR)	8.0 (5.0–28.0)	18.0 (6.0–30.0)	0.180
MV day, day, median (IQR)	9.5 (4.0–20.0)	18.0 (11.0–31.0)	0.003

Data are presented as number (%) or medians (interquartile ranges).

*GI bleeding* gastrointestinal bleeding, *PGD* primary graft dysfunction, *ICU* intensive care unit, *MV* mechanical ventilation.

^a^*P* value determined by *t* test or χ^2^ test.

### Subgroup analysis of AKI characteristics according to the AKI severity and onset

There were no significant differences in baseline characteristics, comorbidities, or intraoperative variables between AKI stage I or II and AKI stage III. Risk factors for the development of AKI, such as anemia, number of RBC transfusion units during surgery, and usage of intravenous colistin infusion, were not significantly different between the two severity groups (Supple Table 1). Also, there were no significant changes in body weight during the post-operative period, indicating that similar management of fluid balance was applied.

We further evaluated AKI according to the timing of AKI occurrence. Early AKI was defined as that within 1 week after LT, late AKI development was classified as that from 1 week to 1 month after LT (Supple Table 2). The incidence of early AKI was 67.7% (n = 40), while that for late AKI was 32.2% (n = 19). The reason for limiting late AKI to just 1-month was because, after one month, the incidence of AKI decreased to 7.4%, and after 1 month, there were no cases of HD or RRT (Supple Table 3). There were no significant differences in age, primary lung diseases, comorbidities, and bridging MV or ECMO between the two AKI timing groups. Also, there were no differences in intra-operative variables, such as intra-operative bleeding, intra-operative fluid intake, and RBC transfusion during surgery between the two groups. Interestingly, development of AKI stage III was more common in the early post-operative period (72.5% vs 21.1%) (Supple Table 2). AKI I or II tended to developed in late post-operative period (78.9% vs. 27.5%). However, Colistin usage, which was a risk factor for AKI development, was similar and reached over 40% in both groups. Overall, body-weight during surgery increased nearly 6.0 kg in the AKI group, and, slowly decreased to near pre-op body -weight within 2 weeks. However, in the early AKI group, the mean body-weight change between POD7 and POD3 was statistically lower than that in the late AKI group. (early AKI vs. late AKI; − 2.65[− 4.55, − 0.50] vs. − 3.70 [− 7.10, − 2.90]; *P* = 0.026] (Supple Table 2). In the early AKI group, the proportion of patients who received RRT due to renal failure was higher than that in the late AKI group (65.0% vs 21.1%, *P* = 0.002) (Supple Table 2).

### AKI and mortality rates after lung transplantation

**Figure 2:**
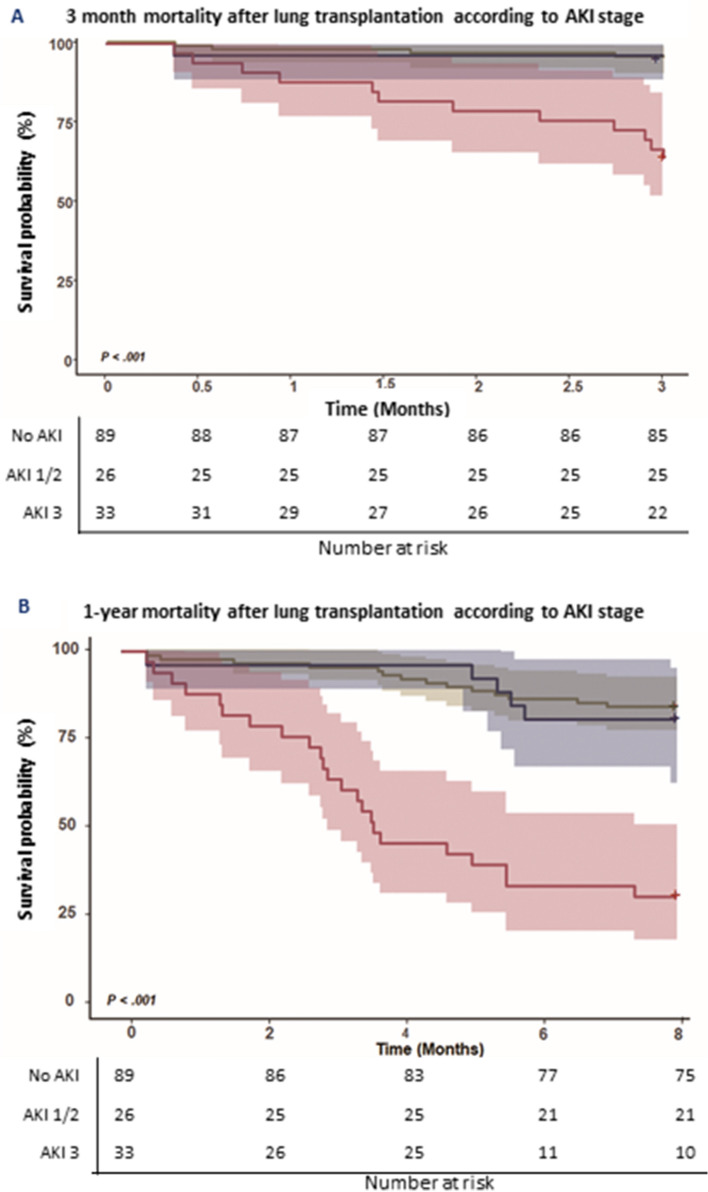
Kaplan–Meier survival analysis of lung transplant recipients stratified by AKI stage: 3-month mortality A, and 1-year mortality B. (**A**) Kaplan–Meier 3-month mortality of lung transplant patients stratified by AKI stage. (**B**) Kaplan–Meier 1-year mortality of lung transplant patients according to AKI stage. *AKI* acute kidney injury.

In Kaplan–Meier curves, the post-operative 3-month mortality rate in patients with no AKI, AKI I or II, and AKI III were 4.5% (4/89), 3.8% (1/26), and 36.4% (12/33), respectively (log-rank test, P < 0.001, Fig. 2A). Patients with no AKI, and those with AKI I or II had no significant difference in 3-month mortality. However, the AKI stage III group had significantly higher mortality.

The 1-year mortality rates in patients with no AKI, AKI I or II, and AKI III were 20.2% (18/89), 34.6% (9/26), and 72.7% (24/33), respectively (log-rank test, *P* < 0.001, Fig. 2B). The more severe AKI became, the higher the 1-year morality rate was. We further compared mortality in the AKI III group according to RRT use, and there was no significant difference in mortality between those who did or did not receive RRT (Supple Table 4). In the univariate Cox proportional model used for analyzing 1-year mortality, age, albumin, number of RBC transfusion units, and sepsis were risk factors for mortality after LT. In multivariable analysis, age and AKI were independent risk factors for 1-year mortality (Table 5).

**Table 5:** Cox proportional hazard regression model for 1-year mortality after lung transplantation.

Variables	Univariable		Multivariable	
	HR (95% CI)	*P* value^a^	HR (95% CI)	*P* value^a^
Age, years	1.097 (1.058–1.137)	0.001	1.092 (1.051–1.135)	0.001
BMI, kg/m^2^	1.050 (0.981–1.124)	0.162		
Hb	0.902 (0.796–1.021)	0.102		
Albumin	0.656 (0.455–0.946)	0.024	0.850 (0.559–1.293)	0.447
Pre-op ECMO	1.085 (0.484–2.433)	0.844		
Pre-op MV	1.501 (0.796–2.832)	0.210		
Number of RBC transfusion units	1.067 (1.014–1.123)	0.012	1.030 (0.965–1.099)	0.369
Operation time	4.71 (0.48–46.45)	0.180		
Colistin intravenous infusion	1.640 (0.879–3.060)	0.120		
AKI	3.688 (1.992–6.829)	0.001	2.921 (1.495–5.707)	0.002
PGD 3, 72 h	1.719 (0.826–3.578)	0.148		
Sepsis, within 1 month	2.133 (1.143–3.983)	0.017	1.473 (0.725–2.990)	0.284

*BMI* body mass index, *Hb* hemoglobin, *ECMO* extra corporeal membrane oxygenation, *MV* mechanical ventilation, *RBC* red blood cell, *AKI* acute kidney disease, *PGD* primary graft dysfunction.

^a^*P* value determined by Cox proportional hazard model.

## Discussion

In our study, we focused on the development of AKI within 1 month, a longer follow-up duration than those in previous studies, which primarily focused on AKI at post-operative 72 h or 1 week^4–10,19^. Reflecting the natural course of kidney injury after LT, AKI more often appears to be an epiphenomenon of the pre-, intra-, and post-operative clinical course after LT. A longer duration of follow up would be necessary to analyze post-operative factors, such as using nephrotoxic agents, sepsis, and other post-operative complications that affect AKI.

The incidence of AKI within 1 month after LT defined by AKIN classification was 39% in our cohort, lower than that reported in other studies. This could be explained by the older age and different proportions of primary lung disease compared to other studies. Serum creatinine is usually underestimated in older adult patients due to reduced muscle mass^20^, and thus, changes in sCr levels could have been underestimated in our cohort. In the ISHLT registry report chronic obstructive pulmonary disease (COPD) was the most common indication in the last decade, while idiopathic interstitial pneumonia was the second^21^. However, in our cohort the proportions of IPF and connective-tissue disease related interstitial lung disease were over 60%, while that of COPD was lower than 10%. These differences may explain the lower incidence of AKI development, compared to other studies.

Prior studies have reported on the risk factors of AKI after LT. Ishikawa et al. reported that perioperative risk factors of AKI within 72 h included intraoperative hypoxemia (SpO_2_ < 90%) in patients that underwent double-lung transplantation, compared to those that underwent single-lung transplantation^8^. Balci et al*.* reported on perioperative risk factors of LT within 1 month as intraoperative hypotension and systemic infection in the post-operative period^9^. Amphotericin B use and high tacrolimus level were reported as risk factors of AKI in some studies^10,22^.

The main finding of our study is that pre-operative anemia, number of RBC units transfused during surgery, and usage of colistin intravenous for multi drug resistant (MDR) pathogens independently are correlated with AKI development. Ho et al. reported that in cases of cardiac surgery with cardiopulmonary bypass, nearly all patients are at the ‘initiation phase’ of ischemia–reperfusion kidney injury with proximal tubular dysfunction. They suggested that the ‘extension phase’ of kidney injury is related with the severity of inflammatory response, renal hypoxia, and oxidative stress^23,24^. As CPB, and ECMO are used during LT, similar principals would likely apply. Accordingly, one could be reason that those described risk factors would be variables preventing recovery from initial kidney injury, therby leading to, progressing to AKI.

Underlying anemia has been shown to be associated with prolonged hypoxemia and could result in AKI in the post LT period^25^. Recipients of LT already face respiratory failure due to primary lung disease, which could be further aggravated by anemia, inducing a prolonged hypoxemic status. This reduced oxygen-carrying capacity elicits sympathetic nerve system increases in vascular resistance, causing vasoconstriction and leading to renal hypo-perfusion^26^. Thus, anemia prior to surgery risk for developing AKI in LT recipients.

An increased amount of packed RBC transfusion units can induce transfusion-related adverse effects. Intraoperative RBC requirements are most likely necessary due to intraoperative requirements for hemodynamics, although in our analysis, observed hemodynamic instability during surgery was not statically related to AKI development, despite RBC requirements being statistically higher in the AKI group. Garg et al. reported in a randomized controlled trial, that restrictive transfusion of RBC was not inferior as an outcome of post-operative AKI in cardiac surgery with CPB^24,27^. A unit of RBC is known to be stored up to 42 days and can undergo erythrocyte membrane change, becoming more fragile and leading to progressive hemolysis. Accumulation of pro-inflammatory molecules, free hemoglobin, and iron can lead to the development of AKI^28^.

Among nephrotoxic antibiotics, Intravenous colistin which was used to treat MDR pathogens, was an independent risk factor for developing AKI. Colistin has high intrinsic renal toxicity and accumulates within the proximal cortical tissue. Combined with anemia, age, liver disease, and baseline GFR, colistin has been reported as a risk factor for AKI^29^. Thus, these findings support that LT recipients who already have anemia are prone to AKI after colistin use. In Southeast Asia, an increased incidence of MDR infections among ICU patients has been reported^30^. A similar situation was observed in our study. A total of 54% (n = 80) of the recipients experienced infections with MDR pathogens. The proportions of *Acinetobacter*, *Pseudomonas*, and *Klebsiella* were 30.4% (n = 45), 15.1% (n = 22), and 18.9% (n = 28), respectively.

In patients who developed AKI, post-operative complications of post-operative bleeding, grade 3 PGD, and sepsis occurrence were more common, compared to those without AKI. The cause and effect of AKI, as well as the complications, were unclear because patients with these complications could develop AKI both early and later after LT. However, in our study, it was clear that sepsis and grade 3 PGD were strongly associated with severe AKI, the majority of cases of which occurred within 7 post-operative days, resulting in increased mortality.

In this study, poor respiratory outcomes (prolonged ventilator days and longer ICU stay) were more common in the AKI group. The renal tubular epithelium is a major site of cell injury and death in AKI, where cytokines, oxidative stress, and leukocytes initiate local and systemic inflammation. The lungs, with their large capillary networks, are capable of sequestering a large number of inflammatory/immune cells^31^. Moreover, AKI-induced derangement of nitric oxide synthase and heme oxygenase (key oxidative stress enzymes) may influence lung function^32^. This could lead to post-LT respiratory failure, leading to difficulty in weaning a patient off the ventilator.

Post-operative treatment of transplanted sick patients can be difficult and challenging. In our study, recipients who develop of AKI experience poor post-operative outcomes and mortality. Post-operative AKI could be a marker indicating that LT recipients are not going to do well. Pre-operative anemia, number of RBC transfused units during surgery, and usage of intravenous colistin were independent predictors of the development of AKI.

Managing the difficult course involving AKI could be important in post-operative care of transplanted sick patients. Technical bleeding control and not having an excessive packed RBC transfusion would be considered in individuals susceptible to AKI. Also, in instances where an infection with MDR pathogens is documented within 7 days, waiting to determine whether it is a true infection or contamination may be advisable, and using colistin inhalation rather than colistin infusion could be another option for post op management. Also, if the candidates were in vulnerable for developing many complications such as AKI selection of recipients for lung transplantation would be a better management strategy, considering organ scarcity and predicted survival outcome.

Our study had several limitations. It was single-center, retrospective cohort study, thus generalizability of our findings may be limited. Second, dosage and duration of administered nephrotoxic agents were not fully evaluated due to inadequate information.

## Conclusion

**Figure 3:**
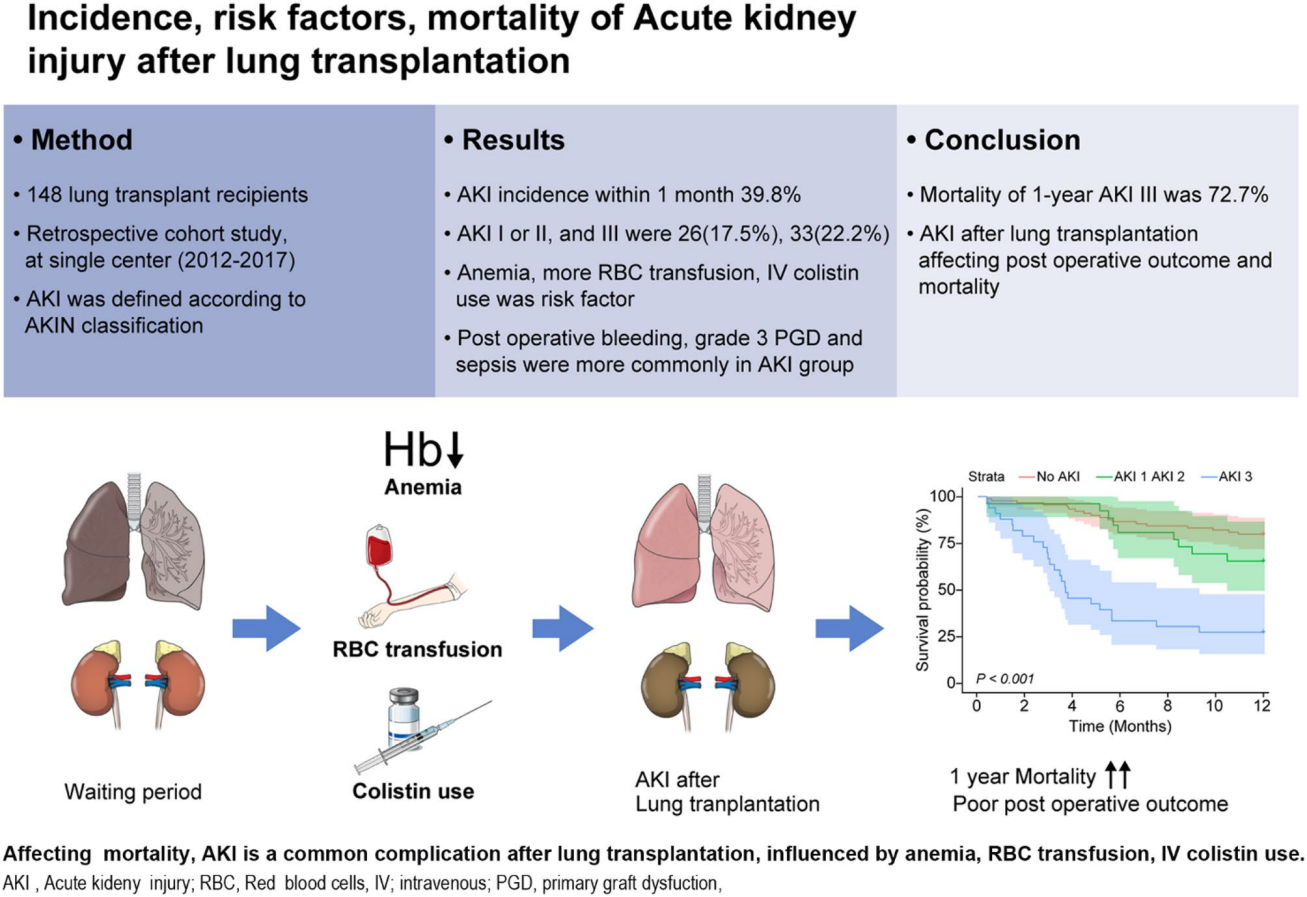
A visual summary in this study. Incidence, risk factors and mortality of acute kidney injury after lung transplantation. During waiting period, LT candidates have end-stage lung disease (gray lung) with normal renal function (brown kidney). Through clinical course of a pre-, intra-, and post-operative process, AKI more often to be appeared with risk factor such as anemia, RBC transfusion and intravenous colistin. Although recipients have a successful new transplanted lung (pink lung), AKI group (dark brown kidney) has higher 1-year mortality with more common complications such as post-operative bleeding, grade 3 PGD, sepsis. As the stage of AKI become severe, the mortality gets higher. Especially AKI with stage III have the highest 1-year mortality, up to 72.7%. We underwent LT to prolong life expectancy of terminal lung disease patients, however recipients with AKI become poorly survive within 1-year, unfortunately. Figure 3 was drawn by MID (Medical Illustration & Design), part of the Medical Research Support Services of Yonsei University College of Medicine. *AKI* acute kidney injury, *RBC* red blood cells, *Hb* hemoglobin, *IV* intravenous, *PGD* primary graft dysfunction.

In post lung transplantation, AKI was developed 39% within one month. The main risk factors for AKI was anemia, higher number of RBC transfusion units during surgery, and intravenous colistin infusion (Fig. 3). AKI was associated with worse post-operative outcome and 3-month, 1-year mortality after LT. Severity of AKI was usually determined in early post op period after LT, so optimal post-operative management as well as recipients selection should be done in sick candidates with highly susceptible to become AKI.

## Supplementary Information

Supplementary Information 1.
