# Supplementary material for: Risk factors and mortality of acute kidney injury within 1 month after lung transplantation
Source: Sci Rep. 2021 Aug 30;11:17399. doi: 10.1038/s41598-021-96889-1 (PMC8405794; doi:10.1038/s41598-021-96889-1)
Supplement: Supplementary file 1 — Supplementary Information 1. [file 41598_2021_96889_MOESM1_ESM.docx]

*Supplemental Table 1. Subgroup Analysis of Postoperative AKI Development according to AKI Severity*

|  | AKI stage I or II  (n=26) | AKI stage III  (n=33) | *p* value^a^ |
| --- | --- | --- | --- |
| Age, years, median (IQR) | 56.5 (44.0-64.0) | 59.0 (52.0-62.0) | 0.685 |
| Male, sex, n (%) | 13 (50.0) | 21 (63.6) | 0.293 |
| BMI, kg/m^2^, median (IQR) | 20.8 (18.7-22.8) | 21.4 (19.2-23.9) | 0.241 |
| Primary lung disease, n (%) |  |  | 0.518 |
| IPF, | 13(50.0) | 13 (39.4) |  |
| CTD-related ILD | 3(11.5) | 7 (21.2) |  |
| AIP | 1(3.8) | 2 (6.1) |  |
| BO after PBSCT | 4(15.4) | 2 (6.1) |  |
| Other* | 5(12.8) | 9 (27.45) |  |
| Comorbid conditions , n (%) |  |  |  |
| Hypertension | 3 (11.5) | 8 (24.2) | 0.214 |
| Diabetes mellitus | 7 (26.9) | 8 (24.2) | 0.814 |
| Pulmonary hypertension | 11 (42.3) | 19 (57.6) | 0.244 |
| Laboratory test, median (IQR) |  |  |  |
| Hb, g/d | 10.75 (8.8-12.7) | 11.1 (9.8-12.7) | 0.783 |
| Cr (baseline), mg/dL | 0.54 (0.49-0.89) | 0.53 (0.38-0.64) | 0.631 |
| eGFR (baseline) | 90.0 (90.0-90.0) | 90.0 (90.0-90.0) | 0.121 |
| Albumin, mg/d | 2.75 (2.40-3.70) | 2.70 (2.40-3.50) | 0.536 |
| Donor variables |  |  |  |
| Donor age, years, median (IQR) | 41.0 (39.0-45.0) | 46.0 (40.0-55.0) | 0.151 |
| Donor sex, n (%) | 14 (53.8) | 21 (63.6) | 0.447 |
| Smoking, pack years, median (IQR) | 0.95 (0.0-10.0) | 2.5 (0.0-15.0) | 0.673 |
| Perioperative variables |  |  |  |
| Pre-op ECMO bridging , n (%) | 6 (23.1) | 8 (24.2) | 0.917 |
| Pre-op MV, n (%) | 9 (34.6) | 14 (42.4) | 0.541 |
| Pre-op APACHE II, median (IQR) | 24.0 (20.0-34.0) | 27.0 (20.0-31.0) | 0.973 |
| Ischemia time, hour, median (IQR) | 299.5 (260.0-318.0) | 290.0 (263.0-340.0) | 0.982 |
| Operation time, hour, median (IQR) | 7.80 (7.00-9.25) | 8.41 (7.45-9.91) | 0.114 |
| Donor-recipient size mismatch, n (%) | 8 (30.8) | 5 (15.2) | 0.151 |
| Number of RBC transfusion units, n, median (IQR) | 6.0 (6.0-10.0) | 8.0 (6.0-13.0) | 0.806 |
| Intra-op fluid intake, ml (IQR) | 8250.0 (5619.5-11325.0) | 7350.0 (6110.0-11550.0) | 0.971 |
| Intra-op output, ml (IQR) | 1285.0 (822.50-2405.0) | 1550.0 (870.0-2630.0) | 0.526 |
| Urine output, POD0, ml(IQR) | 1965.0 (1287.5-2955.0) | 2050.0 (1183.0-2470.0) | 0.549 |
| Body weight |  |  |  |
| Before operation, Kg (IQR) | 53.88 (45.00 – 61.50) | 55.90 (47.90-64.00) | 0.360 |
| POD 0, Kg (IQR) | 59.80 (53.60-67.30) | 62.10 (52.50 -70.20) | 0.536 |
| POD 3, Kg (IQR) | 59.2 (52.70-67.50) | 62.0 (54.60-68.60) | 0.397 |
| POD 7, Kg (IQR) | 54.70 (50.60-67.30) | 60.0 (51.10-68.50) | 0.393 |
| POD 14, Kg (IQR) | 54.0 (44.0-62.0) | 57.0 (50.10-66.50) | 0.151 |
| POD 30, Kg (IQR) | 51.0 (45.20-60.90) | 57.35 (48.50-61.50) | 0.240 |
| Delta Body weight |  |  |  |
| POD 0 – Pre-op, Kg (IQR) | 6.35 (4.50, 8.90) | 5.10 (3.40-9.50) | 0.368 |
| POD 3 – POD0, Kg (IQR) | 0.05 (-1.40, 1.00) | 0.70 (-1.10, 2.30) | 0.261 |
| POD 7 – POD 3, Kg (IQR) | -3.15 (-6.10, -1.40) | -3.10 (-4.70, -0.70) | 0.737 |
| POD 14 – POD 7, Kg (IQR) | -3.80 (-5.00, -0.10) | -2.20 (-5.70, -0.40) | 0.789 |
| POD 30- POD 14, Kg (IQR) | -0.90 (-4.30, 0.80) | -0.10 (-4.30, 1.80) | 0.515 |
| Postoperative antibiotics, n (%) |  |  |  |
| Colistin IV infusion | 11 (42.3) | 17 (51.5) | 0.482 |
| Colistin inhalation | 13 (50.0) | 14 (42.4) | 0.562 |
| Amikacin IV infusion | 3 (11.5) | 8 (13.6) | 0.214 |
| Amphotericin B IV Infusion | 6 (23.1) | 8 (24.2) | 0.917 |
| 3-month mortality | 1 (3.8) | 12 (36.4) | 0.004 |
| 1-year mortality | 9 (34.6) | 24 (72.7) | 0.001 |
| Hemodialysis within one month, n (%) | 0 (0) | 17 (51.5) | 0.001 |
| Renal replacement therapy, n (%) | 0 (0) | 27 (81.8) | 0.001 |

^a^ *p* Value determined by Mann-Whitney U test or χ^₂^ test.

Data are presented as numbers (percentages) or medians (IQR).

BMI: body mass index; IPF: Idiopathic pulmonary fibrosis; CTD: Connective tissue disease; ILD: Interstitial lung disease; AIP: Acute interstitial pneumonia; BO after PBSCT: Bronchiolitis obliterans after peripheral blood stem cell transplantation; eGFR: estimated glomerular filtration rate; Hb: hemoglobin; Cr: Creatine; ECMO: Extra corporeal membrane oxygenation; MV: Mechanical ventilation; RBC: Red blood cell; APACHE: Acute physiology and chronic health evaluation; IV: intravenous; POD: Post-operative day

*Supplemental Table 2. Subgroup Analysis of Postoperative AKI Patients according to Early (within 1 week) and Late (from 1 week to 1 month) AKI Development after Lung Transplantation*

|  | Early developed AKI  (n=40) | Late developed AKI  (n= 19) | *p* value^a^ |
| --- | --- | --- | --- |
| Age, years, median (IQR) | 57.0 (51.0-62.0) | 59.0 (53.5-63.5) | 0.495 |
| Male, sex, n (%) | 21 (52.5) | 13 (68.4) | 0.275 |
| BMI, kg/m^2^, median (IQR) | 20.5 (18.7-24.5) | 21.3 (20.5-22.8) | 0.922 |
| Primary lung disease, n (%) |  |  | 0.446 |
| IPF | 16 (40.0) | 10 (52.6) |  |
| CTD-related ILD | 8 (20.0) | 2 (10.5) |  |
| AIP | 2 (5.0) | 1 (5.3) |  |
| BO after PBSCT | 3 (7.5) | 3 (15.8) |  |
| Other | 11 (17.5) | 3 (15.8) |  |
| Comorbidities, N (%) |  |  |  |
| Hypertension | 7 (17.5) | 4 (21.1) | 0.734 |
| Diabetes mellitus | 7 (17.5) | 8 (13.6) | 0.058 |
| Pulmonary hypertension | 23 (57.5) | 7 (36.8) | 0.170 |
| AKI stage |  |  | 0.001 |
| AKI I or II | 11 (27.5) | 15 (78.9) |  |
| AKI III | 29 (72.5) | 4 (21.1) |  |
| Laboratory test, median (IQR) |  |  |  |
| Hb, g/d | 11.25 (9.9-12.8) | 10.4 (8.45-11.6) | 0.181 |
| Cr (baseline), mg/dL | 0.5 (0.3-0.6) | 0.4 (0.3-0.6) | 0.994 |
| eGFR (baseline) | 90.0 (90.0-90.0) | 90.0 (90.0-90.0) | 0.573 |
| Albumin, mg/d | 2.75 (2.4-3.7) | 2.70 (2.40-3.50) | 0.673 |
| Donor variables |  |  |  |
| Donor age, years, median (IQR) | 44.5 (37.5-53.5) | 44.0(29.5-49.5) | 0.673 |
| Donor sex, n (%) | 20 (50.0) | 15 (78.9) | 0.048 |
| Smoking, pack years, median (IQR) | 0.05 (0.0-15.0) | 2.0 (0.0-11.25) | 0.463 |
| Perioperative variables |  |  |  |
| Pre-op ECMO bridging , n (%) | 9 (22.5) | 5 (26.3) | 0.753 |
| Pre-op MV, n (%) | 15 (37.5) | 8 (42.1) | 0.780 |
| pre-op APACHE II score, median (IQR) | 26.5 (19.0-33.0) | 22.0 (21.0-29.5) | 0.550 |
| Ischemia time, hour, median (IQR) | 287.5 (253.5-343.0) | 312.0 (276.5 -323.5) | 0.390 |
| Operation time, hour, median (IQR) | 8.15 (7.27-9.85) | 8.10 (7.12-9.32) | 0.516 |
| Donor-recipient size mismatch, n (%) | 6 (15.0) | 7 (36.8) | 0.092 |
| Number of RBC transfusion, median (IQR) | 7.0 (5.0-12.0) | 6.0 (6.0-10.5) | 0.732 |
| Intra-op fluid intake, ml (IQR) | 7875.0 (6150.0-11550.0) | 7350.0 (5440.0-10725.0) | 0.425 |
| Intra-op output, ml (IQR) | 1565.0 (840.0-2900.0) | 1200.0 (890.0 -2095.0) | 0.383 |
| Urine output, POD0, ml (IQR) | 2140.0 (1183.0-2650.0) | 1490.0 (1142.50-2235.0) | 0.354 |
| Body weight |  |  |  |
| before operation, Kg (IQR) | 53.13 (45.25-62.35) | 57.50 (52.55-63.25) | 0.322 |
| POD 0, Kg (IQR) | 60.80 (51.35-68.25) | 62.50 (56.25-70.15) | 0.431 |
| POD 3, Kg (IQR) | 60.8 (52.50-67.95) | 62.50 (56.45-68.85) | 0.581 |
| POD 7, Kg (IQR) | 59.25 (50.05-66.70) | 58.00 (53.20-64.45) | 0.935 |
| POD 14, Kg (IQR) | 55.00 (47.80-63.55) | 56.10 (49.10-64.55) | 0.759 |
| POD 30, Kg (IQR) | 54.33 (44.95-60.40) | 54.50 (49.25-63.25) | 0.513 |
| Delta BW |  |  |  |
| POD 0 – Pre-op, Kg (IQR) | 5.80 (4.10-9.55) | 6.00 (3.00-8.80) | 0.752 |
| POD 3 – POD0, Kg (IQR) | 0.25 (-1.00, 1.95) | 0.50 (-1.50, 1.55) | 0.543 |
| POD 7 – POD 3, Kg (IQR) | -2.65 (-4.55, -0.50) | -3.70 (-7.10, -2.90) | 0.026 |
| POD 14 – POD 7, Kg (IQR) | -2.70 (-5.70, -0.65) | -1.40 (-4.30, 0.50) | 0.117 |
| POD 30- POD 14, Kg (IQR) | -0.35 (-4.60, 1.95) | -0.80 (-3.80, 1.35) | 0.908 |
| Post-op Antibiotics, n (%) |  |  |  |
| Colistin IV infusion | 18 (45.0) | 10 (52.6) | 0.781 |
| Colistin inhalation | 17 (42.5) | 10 (52.6) | 0.579 |
| Amikacin IV infusion | 8 (20.0) | 3 (15.8) | 0.499 |
| Amphotericin B IV infusion | 12 (30.0) | 2 (10.5) | 0.091 |
| Hemodialysis , n (%) | 16 (40.0) | 5 (26.3) | 0.389 |
| Renal replacement therapy, n (%) | 27 (65.0) | 4 (21.1) | 0.002 |

^a^ *p* Value determined by Mann-Whitney U test or χ^₂^ test.

Data are presented as numbers (percentages) or medians (IQR)

BMI = body mass index; IPF = Idiopathic pulmonary fibrosis; CTD = Connective tissue disease; ILD = Interstitial lung disease; AIP = Acute interstitial pneumonia; BO after PBSCT = Bronchiolitis obliterans after peripheral blood stem cell transplantation; eGFR = estimated glomerular filtration rate; Hb = hemoglobin; Cr = Creatine; ECMO = Extra corporeal membrane oxygenation; MV = Mechanical ventilation; RBC = Red blood cell; APACHE = Acute physiology and chronic health evaluation; IV = intravenous; POD = Post-operative day.

*Supplemental Table 3. AKI incidence during post-operative period*

|  | N (%) | HD (%) | RRT (%) |
| --- | --- | --- | --- |
| Early AKI (within POD 7) | 40 (27.0) | 16 (10.8) | 26 (17.5) |
| Late AKI (from POD 7 to 1 month) | 19 (12.8) | 5 (3.3) | 4 (2.7) |
| AKI after 1 month within 6 month | 11 (7.4) | 0 (%) | 0 (%) |

Data are presented as numbers (percentages)

HD = hemodialysis; RRT = renal replacement therapy.

*Supplemental Table 4. Mortality rate between AKI III with RRT and without RRT*

|  | AKI III with RRT (N = 27) | AKI III without RRT (N = 6) | *p* value^a^ |
| --- | --- | --- | --- |
| 3-month mortality | 0 (0) | 12 (44.4) | 0.064 |
| 1-year mortality | 4 (66.7) | 20 (74.1) | 0.350 |

^a^ *p* Value determined by χ^₂^ test.

Data are presented as numbers (percentages).

AKI = acute kidney injury; RRT = Renal replacement therapy.
